# Prenatal Alcohol Exposure and Congenital Heart Defects: Retinoic Acid Deficiency as a Potential Mechanism in Dextro-Type Transposition of the Great Arteries

**DOI:** 10.3390/pathophysiology32030035

**Published:** 2025-07-10

**Authors:** Roberto Paparella, Carolina Putotto, Marco Fiore, Fiorenza Colloridi, Paolo Versacci, Mauro Ceccanti, Bruno Marino, Luigi Tarani

**Affiliations:** 1 Department of Maternal Infantile and Urological Sciences, Sapienza University of Rome, 00185 Rome, Italy; 2 Institute of Biochemistry and Cell Biology (IBBC-CNR), Department of Sensory Organs, Sapienza University of Rome, 00185 Rome, Italy; 3 Società Italiana per il Trattamento dell’Alcolismo e le sue Complicanze (SITAC), 00185 Rome, Italy

**Keywords:** fetal alcohol spectrum disorder, congenital heart defects, prenatal alcohol exposure, transposition of the great arteries, retinoic acid, cardiovascular disorders

## Abstract

Fetal alcohol spectrum disorder (FASD) is a preventable cause of developmental disabilities linked to prenatal alcohol exposure (PAE). Congenital heart defects (CHDs) are frequently observed in FASD, with a notable association between PAE and dextro-type transposition of the great arteries (d-TGA). A potential pathogenetic mechanism of d-TGA in FASD, involving retinoic acid (RA) deficiency due to the interference of ethanol with RA biosynthesis, is proposed. Further investigation is required to understand the timing and impact of alcohol exposure on congenital anomalies, particularly in the context of CHDs.

## 1. Introduction

Fetal alcohol spectrum disorder (FASD) is a term that is used to describe the wide range of developmental defects and neurobehavioral anomalies due to the teratogenic effects of alcohol taken during pregnancy [1,2,3,4]. A safe threshold of the amount or time of alcohol consumption during pregnancy has not been identified. Therefore, it is recommended that women completely avoid drinking alcohol while pregnant or trying to conceive, since FASD is the most common, totally avoidable, and preventable cause of acquired intellectual disability in childhood [5,6].

Congenital heart defects (CHDs), which represent the major cause of infant morbidity and birth defect-related deaths, are the most common type of congenital anomalies, with an overall reported prevalence of approximately 10 per 1000 live births. CHDs have been frequently reported in FASD [6,7]. Apart from genetic causes, the pathogenesis of CHDs is still largely unknown. However, several environmental factors are associated with an increased risk of CHDs, including prenatal alcohol exposure (PAE) [8,9,10]. A recent meta-analysis showed that PAE was not associated with particular subtypes of CHDs, except for a statistically significant association found with a specific CHD: dextro-type transposition of the great arteries (d-TGA), a cyanotic heart defect characterized by atrioventricular concordance and ventriculo-arterial discordance [11].

Despite the increasing recognition of this association, the precise mechanisms by which ethanol exposure contributes to d-TGA remain poorly understood. Emerging evidence suggests that ethanol disrupts key developmental signaling pathways, including retinoic acid (RA) metabolism, which plays a crucial role in cardiac morphogenesis. Ethanol exposure leads to RA deficiency by inhibiting the activity of retinaldehyde dehydrogenases (RALDHs), the enzymes responsible for RA synthesis [12,13]. This deficiency may impair the normal differentiation of cardiac progenitor cells, particularly those in the second heart field (SHF), which contribute to the development of the great arteries and outflow tract. Experimental models have also indicated that ethanol-mediated RA deficiency down-regulates key transcription factors implicated in left–right patterning and proper cardiac septation. This interference may therefore contribute to the embryological abnormalities leading to d-TGA, given the established role of RA in cardiovascular development [14]. Additionally, maternal factors such as nutritional status, metabolic conditions, and genetic predisposition may interact with PAE to modulate the risk of d-TGA, necessitating further epidemiological and mechanistic studies to delineate these complex interactions.

Therefore, based on our recent experience with a 4-year-old child diagnosed with FASD—clinically confirmed according to the criteria established by Hoyme et al. [15,16] and d-TGA—we propose a potential pathogenetic hypothesis linking RA disruption to d-TGA in the context of FASD. The patient underwent an arterial switch operation (ASO) in the neonatal period after an echocardiographic diagnosis of d-TGA with an intact ventricular septum. Notably, there was no familial recurrence of CHDs, and the child exhibited no extracardiac anomalies. At present, the patient’s cardiac status remains stable, with regular follow-up evaluations. These findings reinforce the need to further investigate the role of RA deficiency in conotruncal heart defects and consider the possibility that some molecular mechanisms associated with d-TGA overlap with those regulating left–right patterning, even in the absence of overt laterality defects such as dextrocardia, atrial isomerism, or visceral heterotaxy. This opinion paper specifically focuses on d-TGA, exploring its potential pathogenetic link with RA deficiency in the context of FASD.

## 2. Fetal Alcohol Spectrum Disorder (FASD)

The phenotypic spectrum of FASD is influenced by multiple factors, including the dose, frequency, and timing of alcohol consumption during pregnancy, as well as maternal and fetal genetic susceptibility [17]. Due to the lack of a genetic test, since FASD has an epigenetic basis, and the absence of specific and standardized biomarkers for identifying affected individuals, diagnosing FASD remains particularly difficult for clinicians [18,19,20,21]. The effects of PAE range from the absence of damage to mortality, depicting a continuum of highly variable outcomes. The most severe condition is called fetal alcohol syndrome (FAS), which refers to individuals who show cardinal facial features (short palpebral fissures, thin upper lip, smooth philtrum), neurobehavioral deficits, and growth retardation [15]. In addition to FAS, FASD includes partial FAS (pFAS), alcohol-related birth defects (ARBDs), and alcohol-related neurodevelopmental disorder (ARND) [22]. A neurobehavioral disorder associated with prenatal alcohol exposure (ND-PAE) was introduced in the Diagnostic and Statistical Manual of Mental Disorders, Fifth Edition (DSM-5) to indicate the neurobehavioral consequences of PAE in the absence of growth retardation or facial dysmorphisms in a similar, but not completely overlapping, way to ARND [23]. A variety of structural congenital defects and other anomalies may therefore be due to PAE, as well as the salient, characteristic findings of FASD [24].

## 3. A Brief Overview of Congenital Heart Defects (CHDs)

CHDs represent the most prevalent class of congenital anomalies, affecting approximately 1% of live births worldwide [7]. They are a leading cause of infant morbidity and mortality, accounting for nearly 30% of all deaths related to congenital anomalies in the neonatal period [25]. The etiology of CHDs is multifactorial, involving a complex interplay between genetic predisposition, environmental influences, and maternal health factors. While significant progress has been made in identifying key genetic contributors, the role of prenatal environmental exposures—such as maternal diabetes, obesity, infections, and teratogenic substances including alcohol—remains an area of active investigation [26].

One of the major environmental risk factors for CHDs is PAE, which has been implicated in several cardiac malformations. As reported in a recent meta-analysis, alcohol consumption during pregnancy leads to a statistically significant increase in risk of conotruncal anomalies and d-TGA [11]. The teratogenic effects of alcohol on fetal heart development are thought to be mediated through mechanisms such as oxidative stress, apoptosis, and disruption of RA signaling, which plays a crucial role in the formation of cardiac structures [12].

Advances in fetal echocardiography and genetic screening have improved early detection and management of CHDs. However, long-term outcomes for affected individuals vary significantly depending on defect severity, timing of cardiac surgery, and presence of comorbid conditions. Many children with complex CHDs require multiple surgeries, lifelong cardiology follow-up, and neurodevelopmental support due to the risk of associated cognitive and motor deficits [27]. Early intervention and multidisciplinary care, including cardiologists, geneticists, neonatologists, and developmental specialists, are essential to optimizing outcomes for these patients.

Since the understanding of CHD pathogenesis continues to evolve, future research should focus on identifying gene–environment interactions, epigenetic modifications, and novel therapeutic approaches to mitigate the impact of teratogenic exposures like alcohol. Additionally, public health initiatives aimed at reducing maternal risk factors, promoting prenatal care, and improving CHD awareness will be critical in decreasing the burden of CHD globally.

## 4. FASD and d-TGA

PAE may act as a contributing factor in the pathogenesis of specific CHDs. The meta-analysis by Yang and colleagues investigated the relationship between PAE and the risk of overall CHDs as well as the presence of specific CHDs, indicating a statistically significant association with d-TGA (OR = 1.64, 95% CI = 1.17–2.30) [11]. Two other studies have explored the effects of PAE on the d-TGA; however, the underlying mechanisms by which PAE may contribute to this specific cardiac anomaly remain poorly understood [9,10]. The d-TGA is one of the most common cyanotic CHDs, with approximately 1 in 3500–5000 live births [28]. It is characterized by discordant ventriculo-arterial connections, in which the aorta arises from the right ventricle and the pulmonary artery arises from the left ventricle. This means that the systemic and pulmonary circulations are in parallel and not in series, preventing oxygenated blood from reaching the systemic circulation, leading to severe hypoxemia shortly after birth [29]. Without intervention, d-TGA is lethal in the neonatal period, but surgical advances, particularly the ASO, have significantly improved survival. Performed within the first weeks of life, ASO restores normal circulation by switching the aorta and pulmonary artery to their correct positions [29]. Long-term outcomes are generally favorable with proper follow-up; however, complications such as arrhythmias, ventricular dysfunction, coronary artery issues, as well as neo-aortic valve insufficiency and root dilatation, may occur over time [30].

The etiology of this CHD is still not fully delineated, but new insights into the pathogenesis were recently reported [31]. While the exact embryological disruptions leading to d-TGA remain under investigation, it is plausible that early perturbations in cardiac looping and neural crest cell migration contribute to the abnormal vessel positioning characteristic of this defect [31]. Further research into the precise molecular mediators, including potential epigenetic modifications triggered by PAE, could provide deeper insights into its pathogenic effects on cardiac development.

### Retinoic Acid Deficiency as a Mechanism in d-TGA Pathogenesis

**Figure 1:**
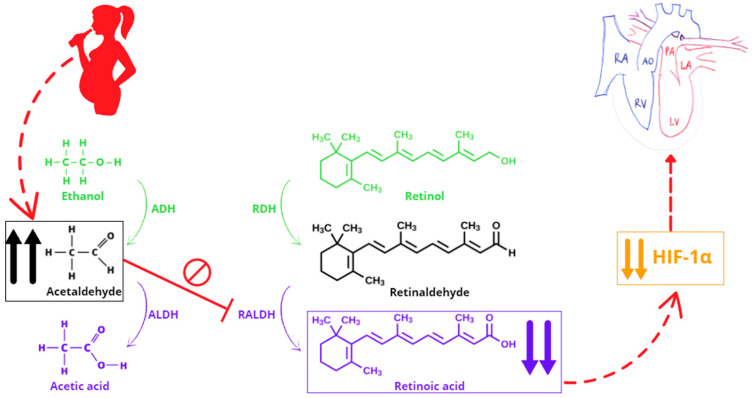
The pathogenetic mechanism linking prenatal alcohol exposure to dextro-type transposition of the great arteries via retinoic acid deficiency. Abbreviations: ADH, alcohol dehydrogenase; ALDH, aldehyde dehydrogenase; AO, aorta; HIF-1α, hypoxia-inducible factor-1α; LA, left atrium; LV, left ventricle; PA, pulmonary artery; RA, right atrium; RALDH, retinaldehyde dehydrogenase; RDH, retinol dehydrogenase; RV, right ventricle. ↑↑ indicates elevation. ↓↓ indicates reduction.

We suggest that a possible explanation of the association between PAE and this specific CHD lies in the teratogenic effect derived from a RA deficiency [Figure 1]. An excessive amount of ethanol (or acetaldehyde, its clearance metabolite) can interfere with RA biosynthesis, since the same families of enzymes are involved in both ethanol clearance and RA production, generating teratogenic consequences [13].

Ethanol consumption during pregnancy disrupts multiple developmental pathways, with one of the most critical being the RA signaling pathway. RA, the active metabolite of vitamin A, plays a fundamental role in embryogenesis, particularly in the development of the cardiovascular system. The teratogenic effects of ethanol are primarily mediated by its metabolism into acetaldehyde and reactive oxygen species, which generate oxidative stress and impair crucial developmental processes [32]. One of the key molecular disruptions caused by ethanol is the inhibition of RALDHs, the enzymes responsible for converting retinaldehyde into RA [33]. This inhibition leads to a localized deficiency in RA in embryonic tissues, particularly affecting the SHF, a progenitor cell population responsible for forming the outflow tract, right ventricle, and great arteries. d-TGA, characterized by a discordant connection between the great arteries and the ventricles, results from defects in outflow tract septation and rotation. RA deficiency impairs the migration and differentiation of cardiac neural crest cells, which contribute to the septation of the truncus arteriosus. Ethanol-induced RA disruption leads to hypoplasia of the aorticopulmonary septum, failing to properly divide the pulmonary artery and aorta. Additionally, reduced RA signaling alters the left–right patterning of the heart, which may contribute to the aberrant positioning of the great arteries seen in d-TGA [14].

These findings are corroborated by experimental studies indicating the reproduction of d-TGA after administration of a RA antagonist in pregnant mice [12], probably due to the HIF-1α (hypoxia-inducible factor-1α) down-regulation following the block of RA [14], and the lower incidence of CHDs whether the HIF-1α expression level is re-established in response to folic acid and methionine supplementation [34].

The observed down-regulation of HIF-1α at both the mRNA and protein levels following RA deficiency, along with its subsequent recovery upon folic acid supplementation, highlights the crucial role of HIF-1α in heart development [14,34]. The localization of HIF-1α in the cardiac primordia further supports its involvement in congenital heart malformations. Given that HIF-1α and its downstream target (e.g., Cited2) are essential for left–right patterning, their dysregulation may contribute to structural heart defects, including d-TGA. A recent overview of the human genetics of d-TGA has highlighted multiple susceptibility loci and candidate genes, further supporting a complex and multifactorial etiology [35]. Since mutations or altered expression of the aforementioned genes are known to cause defects in organ lateralization, these findings reinforce the hypothesis that d-TGA may share certain molecular features with conditions involving disrupted left–right patterning [14,31]; d-TGA may lie at the intersection between laterality defects and conotruncal malformations, involving genes typically associated with left–right axis specification [36].

Moreover, recent findings from animal models have elucidated specific molecular pathways through which disruptions in anterior heart field development may lead to d-TGA, particularly in relation to the altered RA signaling [37]. The possible role of RA deficiency in d-TGA pathogenesis, in the context of FASD, is also strengthened by the available evidence about how the RA signaling contributes to the induction of microcephaly and craniofacial malformations as part of the FASD etiology [38]. The timing of alcohol exposure is one of the most essential factors to be considered, since the interference of PAE on retinoic acid synthesis during early embryogenesis is accountable for the facial features seen in FASD [39]. Furthermore, both in FASD and in RA deficiency, a greater incidence of miscarriages and stillbirths is observed, probably implying a shared signaling pathway [38].

## 5. Conclusions

FASD is a major preventable cause of congenital abnormalities and developmental disabilities. Much needs to be done to explore the effects of alcohol at various stages of gestation, as well as to expand the knowledge about the relationship between PAE and CHD subtypes. We suggest that the specific association between PAE and d-TGA is attributed to the teratogenic effect of RA deficiency resulting from excessive ethanol exposure during early embryogenesis. Identifying the precise molecular mechanisms linking PAE to d-TGA could lead to targeted interventions aimed at mitigating the teratogenic effects of alcohol. Future research should also focus on public health initiatives to reduce maternal alcohol consumption, particularly in high-risk populations. Improved screening, early diagnosis, and multidisciplinary management are essential to optimizing outcomes for children affected by both FASD and CHDs.

## Data Availability

Not applicable.

## Abbreviations

The following abbreviations are used in this manuscript:

ASO	Arterial switch operation
CHDs	Congenital heart defects
d-TGA	Dextro-type transposition of the great arteries
FASD	Fetal alcohol spectrum disorder
HIF-1α	Hypoxia-inducible factor-1α
PAE	Prenatal alcohol exposure
SHF	Second heart field
RALDHs	Retinaldehyde dehydrogenases

## References

[1] del Campo M., Jones K.L. (2017). A Review of the Physical Features of the Fetal Alcohol Spectrum Disorders. Eur. J. Med. Genet..

[2] Abbott C.W., Rohac D.J., Bottom R.T., Patadia S., Huffman K.J. (2018). Prenatal Ethanol Exposure and Neocortical Development: A Transgenerational Model of FASD. Cereb. Cortex.

[3] Brown J.M., Trnka A., Harr D., Dodson K.D., Wartnik H.A.P., Donaldson K. (2018). Fetal Alcohol Spectrum Disorder (FASD): A Beginner’s Guide for Mental Health Professionals. Neurol. Clin. Neurosci..

[4] Hanlon-Dearman A.C., Longstaffe S. (2023). Clinical Perspectives on the Diagnostic Assessment of Individuals with FASD. Neurodevelopmental Pediatrics: Genetic and Environmental Influences.

[5] Williams J.F., Smith V.C. (2015). The COMMITTEE ON SUBSTANCE ABUSE Fetal Alcohol Spectrum Disorders. Pediatrics.

[6] Burd L., Deal E., Rios R., Adickes E., Wynne J., Klug M.G. (2007). Congenital Heart Defects and Fetal Alcohol Spectrum Disorders. Congenit. Heart Dis..

[7] van der Linde D., Konings E.E.M., Slager M.A., Witsenburg M., Helbing W.A., Takkenberg J.J.M., Roos-Hesselink J.W. (2011). Birth Prevalence of Congenital Heart Disease Worldwide. J. Am. Coll. Cardiol..

[8] Harvey D.C., Baer R.J., Bandoli G., Chambers C.D., Jelliffe-Pawlowski L.L., Kumar S.R. (2022). Association of Alcohol Use Diagnostic Codes in Pregnancy and Offspring Conotruncal and Endocardial Cushion Heart Defects. J. Am. Heart Assoc..

[9] Carmichael S.L., Shaw G.M., Yang W., Lammer E.J. (2003). Maternal Periconceptional Alcohol Consumption and Risk for Conotruncal Heart Defects. Birth Defects Res. A Clin. Mol. Teratol..

[10] Grewal J., Carmichael S.L., Ma C., Lammer E.J., Shaw G.M. (2008). Maternal Periconceptional Smoking and Alcohol Consumption and Risk for Select Congenital Anomalies. Birth Defects Res. A Clin. Mol. Teratol..

[11] Yang J., Qiu H., Qu P., Zhang R., Zeng L., Yan H. (2015). Prenatal Alcohol Exposure and Congenital Heart Defects: A Meta-Analysis. PLoS ONE.

[12] Cipollone D., Amati F., Carsetti R., Placidi S., Biancolella M., D’Amati G., Novelli G., Siracusa G., Marino B. (2006). A Multiple Retinoic Acid Antagonist Induces Conotruncal Anomalies, Including Transposition of the Great Arteries, in Mice. Cardiovasc. Pathol..

[13] Fainsod A., Abbou T., Bendelac-Kapon L., Edri T., Pillemer G., Chudley A.E., Hicks G.G. (2022). Fetal Alcohol Spectrum Disorder as a Retinoic Acid Deficiency Syndrome. Fetal Alcohol Spectrum Disorder.

[14] Amati F., Diano L., Campagnolo L., Vecchione L., Cipollone D., Bueno S., Prosperini G., Desideri A., Siracusa G., Chillemi G. (2010). Hif1α Down-Regulation Is Associated with Transposition of Great Arteries in Mice Treated with a Retinoic Acid Antagonist. BMC Genom..

[15] Hoyme H.E., Kalberg W.O., Elliott A.J., Blankenship J., Buckley D., Marais A.-S., Manning M.A., Robinson L.K., Adam M.P., Abdul-Rahman O. (2016). Updated Clinical Guidelines for Diagnosing Fetal Alcohol Spectrum Disorders. Pediatrics.

[16] Hoyme H.E., May P.A., Kalberg W.O., Kodituwakku P., Gossage J.P., Trujillo P.M., Buckley D.G., Miller J.H., Aragon A.S., Khaole N. (2005). A Practical Clinical Approach to Diagnosis of Fetal Alcohol Spectrum Disorders: Clarification of the 1996 Institute of Medicine Criteria. Pediatrics.

[17] Menghi M., Micangeli G., Paparella R., Ceccanti M., Coriale G., Ferraguti G., Fiore M., Fiorentino D., Piccioni M.G., Tarani L. (2024). Italian Guidelines for the Diagnosis and Treatment of Fetal Alcohol Spectrum Disorders: Clinical Hallmarks. Riv. Psichiatr..

[18] Micangeli G., Menghi M., Paparella R., Ceccanti M., Coriale G., Fiorentino D., Ferraguti G., Fiore M., Tarani L. (2024). Interdisciplinary Study Groups Sapienza, ISS, ISTAT, AIDEFAD, SITAC, SIFASD, FIMMG-LAZIO, SIPPS, SIMPESV, CIPE Italian Guidelines for the Diagnosis and Treatment of Fetal Alcohol Spectrum Disorders: Diagnostic Criteria. Riv. Psichiatr..

[19] Astley S.J., Bledsoe J.M., Davies J.K., Thorne J.C. (2017). Comparison of the FASD 4-Digit Code and Hoyme et al. 2016 FASD Diagnostic Guidelines. Adv. Pediatr. Res..

[20] Okulicz-Kozaryn K., Maryniak A., Borkowska M., Śmigiel R., Dylag K.A. (2021). Diagnosis of Fetal Alcohol Spectrum Disorders (Fasds): Guidelines of Interdisciplinary Group of Polish Professionals. Int. J. Environ. Res. Public Health.

[21] Bastons-Compta A., Astals M. (2016). Foetal Alcohol Spectrum Disorder (FASD) Diagnostic Guidelines: A Neuropsychological Diagnostic Criteria Review Proposal. J. Neuropsychopharmacol. Ment. Health.

[22] Hagan J.F., Balachova T., Bertrand J., Chasnoff I., Dang E., Fernandez-Baca D., Kable J., Kosofsky B., Senturias Y.N., Singh N. (2016). Neurobehavioral Disorder Associated with Prenatal Alcohol Exposure. Pediatrics.

[23] Doyle L.R., Mattson S.N. (2015). Neurobehavioral Disorder Associated with Prenatal Alcohol Exposure (ND-PAE): Review of Evidence and Guidelines for Assessment. Curr. Dev. Disord. Rep..

[24] Jones K.L., Hoyme H.E., Robinson L.K., del Campo M., Manning M.A., Prewitt L.M., Chambers C.D. (2010). Fetal Alcohol Spectrum Disorders: Extending the Range of Structural Defects. Am. J. Med. Genet. A.

[25] Hoffman J.I.E., Kaplan S. (2002). The Incidence of Congenital Heart Disease. J. Am. Coll. Cardiol..

[26] Gelb B.D. (2004). Genetic Basis of Congenital Heart Disease. Curr. Opin. Cardiol..

[27] Marino B.S., Lipkin P.H., Newburger J.W., Peacock G., Gerdes M., Gaynor J.W., Mussatto K.A., Uzark K., Goldberg C.S., Johnson W.H. (2012). Neurodevelopmental Outcomes in Children with Congenital Heart Disease: Evaluation and Management: A Scientific Statement from the American Heart Association. Circulation.

[28] Stallings E.B., Isenburg J.L., Rutkowski R.E., Kirby R.S., Nembhard W.N., Sandidge T., Villavicencio S., Nguyen H.H., McMahon D.M., Nestoridi E. (2024). National Population-based Estimates for Major Birth Defects, 2016–2020. Birth Defects Res..

[29] Szymanski M.W., Moore S.M., Kritzmire S.M., Thomas A., Goyal A. (2025). Transposition of the Great Arteries. StatPearls.

[30] van der Palen R.L.F., Blom N.A., Kuipers I.M., Rammeloo L.A.J., Jongbloed M.R.M., Konings T.C., Bouma B.J., Koolbergen D.R., Hazekamp M.G. (2021). Long-Term Outcome after the Arterial Switch Operation: 43 Years of Experience. Eur. J. Cardio-Thorac. Surg. Off. J. Eur. Assoc. Cardio-Thorac. Surg..

[31] Unolt M., Putotto C., Silvestri L.M., Marino D., Scarabotti A., Massaccesi V., Caiaro A., Versacci P., Marino B. (2013). Transposition of Great Arteries: New Insights into the Pathogenesis. Front. Pediatr..

[32] Crabb D.W., Matsumoto M., Chang D., You M. (2004). Overview of the Role of Alcohol Dehydrogenase and Aldehyde Dehydrogenase and Their Variants in the Genesis of Alcohol-Related Pathology. Proc. Nutr. Soc..

[33] Shabtai Y., Fainsod A. (2018). Competition between Ethanol Clearance and Retinoic Acid Biosynthesis in the Induction of Fetal Alcohol Syndrome. Biochem. Cell Biol..

[34] Cipollone D., Carsetti R., Tagliani A., Rosado M.M., Borgiani P., Novelli G., D’Amati G., Fumagalli L., Marino B., Businaro R. (2009). Folic Acid and Methionine in the Prevention of Teratogen-Induced Congenital Defects in Mice. Cardiovasc. Pathol..

[35] Houyel L., Rickert-Sperling S., Kelly R.G., Haas N. (2024). Human Genetics of D-Transposition of Great Arteries. Congenital Heart Diseases: The Broken Heart.

[36] De Ita M., Cisneros B., Rosas-Vargas H. (2021). Genetics of Transposition of Great Arteries: Between Laterality Abnormality and Outflow Tract Defect. J. Cardiovasc. Transl. Res..

[37] Gill E., Bamforth S.D., Rickert-Sperling S., Kelly R.G., Haas N. (2024). Molecular Pathways and Animal Models of D-Transposition of the Great Arteries. Congenital Heart Diseases: The Broken Heart.

[38] Petrelli B., Bendelac L., Hicks G.G., Fainsod A. (2019). Insights into Retinoic Acid Deficiency and the Induction of Craniofacial Malformations and Microcephaly in Fetal Alcohol Spectrum Disorder. Genesis.

[39] Petrelli B., Weinberg J., Hicks G.G. (2018). Effects of Prenatal Alcohol Exposure (PAE): Insights into FASD Using Mouse Models of PAE. Biochem. Cell Biol..

